# Th2 Regulation of Viral Myocarditis in Mice: Different Roles for TLR3 versus TRIF in Progression to Chronic Disease

**DOI:** 10.1155/2012/129486

**Published:** 2011-10-15

**Authors:** Eric D. Abston, Michael J. Coronado, Adriana Bucek, Djahida Bedja, Jaewook Shin, Joseph B. Kim, Eunyong Kim, Kathleen L. Gabrielson, Dimitrios Georgakopoulos, Wayne Mitzner, DeLisa Fairweather

**Affiliations:** ^1^Department of Environmental Health Sciences, Johns Hopkins University Bloomberg School of Public Health, 615 N. Wolfe Street, Baltimore, MD 21205, USA; ^2^Department of Pathology, Johns Hopkins University School of Medicine, 615 N. Wolfe Street, Baltimore, MD 21205, USA; ^3^CVRx Inc., 9201 West Broadway Avenue, Minneapolis, MN 55445, USA

## Abstract

Viral infections are able to induce autoimmune inflammation in the heart. Here, we investigated the role of virus-activated Toll-like receptor (TLR)3 and its adaptor TRIF on the development of autoimmune coxsackievirus B3 (CVB3) myocarditis in mice. Although TLR3- or TRIF-deficient mice developed similarly worse acute CVB3 myocarditis and viral replication compared to control mice, disease was significantly worse in TRIF compared to TLR3-deficient mice. Interestingly, TLR3-deficient mice developed an interleukin (IL)-4-dominant T helper (Th)2 response during acute CVB3 myocarditis with elevated markers of alternative activation, while TRIF-deficient mice elevated the Th2-associated cytokine IL-33. Treatment of TLR3-deficient mice with recombinant IL-33 improved heart function indicating that elevated IL-33 in the context of a classic Th2-driven response protects against autoimmune heart disease. We show for the first time that TLR3 versus TRIF deficiency results in different Th2 responses that uniquely influence the progression to chronic myocarditis.

## 1. Introduction

Heart failure (HF) is the end consequence of a number of cardiovascular diseases including myocarditis and dilated cardiomyopathy (DCM). In spite of advances in diagnosis and treatment, HF remains a growing medical problem associated with major hospitalization, mortality, and poor prognosis [1]. Myocarditis is an autoimmune disease that is responsible for around half of all DCM cases in the United States [2–4]. A recent long-term study of myocarditis patients revealed that inflammation was the best predictor for the progression to HF following acute myocarditis [5]. Viruses like coxsackievirus B3 (CVB3) are often detected in patient myocardial biopsies [3, 6], and antiviral treatments such as interferon (IFN)-*β* reduce inflammation and HF in animal models and patients [7, 8], implying that viral infections are an important cause of myocarditis cases that lead to HF. Viral infections including CVB3 are able to induce autoimmune myocarditis that progresses to DCM in susceptible strains of mice [9, 10].

Toll-like receptor (TLR)3 binds to double-stranded RNA and inhibits viral replication by upregulating IFNs [11, 12]. TLR3 and TLR4 uniquely signal through TIR domain-containing adaptor protein-inducing IFN-*β* (TRIF) at the endosomal surface [12]. Recently, TLR3 polymorphisms in a patient population were associated with an increased occurrence of viral myocarditis and DCM [13]. Previously, TLR3- or TRIF-deficient mice were found to develop increased viral replication and acute myocarditis [11, 14]. Because TRIF participates in TLR3 and TLR4 signaling and TLR3 is known to protect against CVB3 myocarditis [14] while TLR4 increases disease [15], we were interested in determining whether TLR3- and TRIF-deficient mice developed similar disease.

To examine the effect of TLR3 versus TRIF deficiency on myocarditis, DCM, and HF, we used an autoimmune model of CVB3 myocarditis where mice receive infectious virus and heart proteins [9, 16]. We found that TLR3-deficient mice developed a classic T helper (Th)2 response with increased interleukin (IL)-4 and markers of alternative activation during acute CVB3 myocarditis, while TRIF-deficient mice did not. Although both knockout strains developed similarly worse acute CVB3 myocarditis and viral replication compared to wild-type (WT) mice, disease was significantly worse in the absence of TRIF compared to TLR3. Our results indicate that TLR3 protects against CVB3 myocarditis by increasing IFN-*γ* and decreasing a classic IL-4-driven Th2 response, while TRIF protects by increasing IFN-*β* and decreasing the Th2-associated cytokine IL-33. Treatment of WT mice with recombinant (r)IL-33 increased CVB3 myocarditis and impaired cardiac function. However, in the context of an IL-4-skewed Th2 response (i.e., TLR3−/−), rIL-33 treatment prevented cardiac dysfunction. We show for the first time that TLR3 versus TRIF deficiency results in different Th2 responses that uniquely influence the progression to chronic myocarditis.

## 2. Materials and Methods

### 2.1. Experimental Model

Wild-type C57BL/6 (BL/6), B6.129, TLR3-(B6;   129S1-**T** **l** **r**3^**tm**1**Flv**^/**J**)-deficient (TLR3−/−) and TRIF-(C57BL/6-**T** **i** **c** **a** **m**1^**Lps**2^/**J**)-deficient (TRIF−/−) mice were obtained from The Jackson Laboratory (Bar Harbor, ME). Mice were maintained under pathogen-free conditions in the animal facility at Johns Hopkins School of Medicine, and approval was obtained from the Animal Care and Use Committee of the Johns Hopkins University for all procedures. CVB3 (Nancy strain) was obtained from the American Type Culture Collection (ATCC, VA), grown in Vero cells (ATCC) and passaged through the heart as described previously [16]. Mice inoculated ip with uninfected cardiac tissue supernatant diluted in PBS, or PBS alone, do not develop myocarditis (data not shown). Eight-to-ten-week-old male mice were inoculated with 10^3^ PFU of heart-passaged CVB3 containing infectious virus and heart tissue diluted in sterile PBS or PBS alone ip and tissues collected at day 10 (acute myocarditis) or day 35 (chronic myocarditis) pi as described previously [16]. Recombinant rIL-33 (1 *μ*g/0.1 mL, Cat#3626-ML) from R&D Systems (Minneapolis, MN) was diluted in sterile PBS, or PBS only was injected ip on days 1, 3, 5, 7, and 9 pi following CVB3 injection on day 0. All experiments were conducted three or more times with 7 to 12 mice per group except for rIL-33 treatment of TLR3-deficient mice.

### 2.2. Histology

Hearts were fixed in 10% buffered formalin and stained with haematoxylin and eosin (H&E) to assess inflammation. Myocarditis was assessed as the percentage of the heart section with inflammation compared to the overall size of the heart section using a microscope eyepiece grid according to [15]. The development of DCM was assessed by gross observation of histology sections at low magnification and by pressure-volume relationships, as previously described [17, 18].

### 2.3. Cardiac Function

Cardiac function was assessed by pressure-volume catheter (1.2F Scisense Inc., London, ON) placed in the left ventricle via the apex in open-chest mice anesthetized with 3% isoflurane (Baxter, Deerfield, IL), as previously described [19, 20].

### 2.4. Plaque Assay

Hearts from individual mice were homogenized at 10% weight/volume in 2% minimal essential medium (MEM) (MediaTech, Manassas, VA) and individual supernatants used in plaque assays to determine the level of infectious virus, as previously described [15]. Virus levels are expressed as the mean plaque-forming unit (PFU)/g tissue ± standard error of mean (SEM), and the limit of detection is 10 PFU/g of tissue.

### 2.5. ELISA

Hearts were homogenized at 10% weight/volume in 2% MEM and individual supernatants used in ELISA [15, 17]. Cytokines were determined in homogenized supernatants using R&D Systems ELISA kits (Minneapolis, MN), according to the manufacturer's instructions. Levels were expressed as pg/g of heart tissue ± SEM.

### 2.6. RNA Extraction and qRT-PCR

Hearts and spleens were harvested and flash frozen in liquid nitrogen and stored at −80°C. Tissues were homogenized in 2 mL TRIzol (Invitrogen, Carlsbad, CA) according to the manufacturer's protocol. The PureLink Micro-to-Midi Total RNA Purification System (Invitrogen) was used for extraction and purification of RNA. RNA was quantified using a NanoDrop spectrophotometer and quality assessed by RNA Nano LabChip analysis on an Agilent BioAnalyzer 2100 (Agilent Technologies, Santa Clara, CA). Total RNA from hearts or spleens was assessed by quantitative real-time (qRT) PCR using Assay-on-Demand primers and probe sets and the ABI 7000 Taqman System from Applied Biosystems (Carlsbad, CA). Data were normalized to hypoxanthine phosphoribosyltransferase 1 (HPRT) according to [21]. There was no significant difference in HPRT expression in the heart or spleen before or after infection. The mRNA data are presented as a relative gene expression (RGE). RGE is calculated as the ratio of target gene expression (fold change of mRNA of interest) to the normalization control gene expression (fold change of normalization control mRNA).

### 2.7. Statistical Analysis

Two-group analysis of normally distributed data was performed by Student's *t*-test. The Mann-Whitney *U*-test was used to evaluate nonparametric data comparing two groups. Multiple comparisons were analyzed by ANOVA with a Bonferroni correction. A value of *P* < 0.05 was considered significant.

## 3. Results

### 3.1. Inflammation and Viral Replication Increase in a Similar Manner in TLR3- or TRIF-Deficient Mice during Acute CVB3 Myocarditis

**Figure 1:**
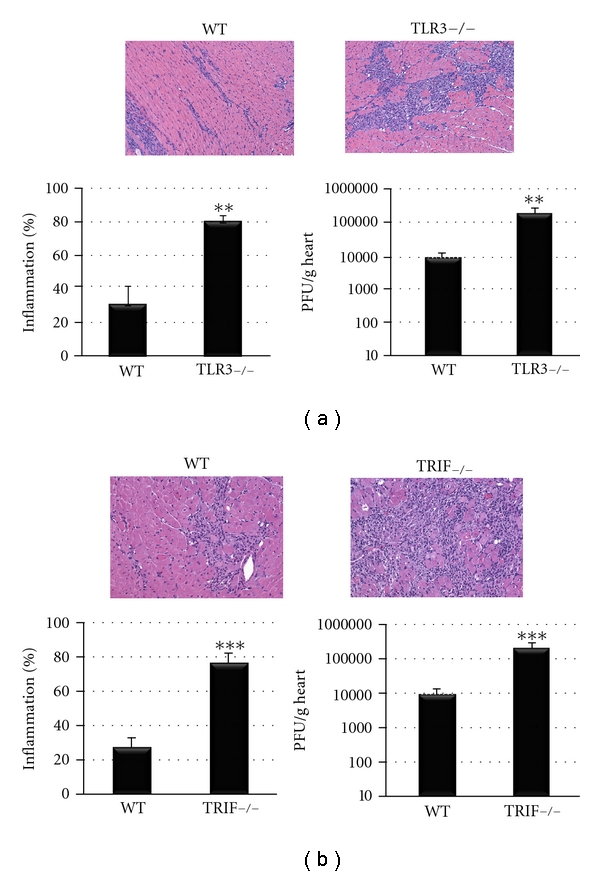
Inflammation and viral replication increase in a similar manner in TLR3-deficient (TLR3−/−) or TRIF−/− mice during acute CVB3 myocarditis. (a) TLR3−/− mice developed increased inflammation and viral replication at day 10 pi compared to WT B6.129 controls. (a) Representative histology sections of inflammation in WT and TLR3−/− hearts stained with H&E (top), magnification ×64. (b) TRIF−/− mice develop increased inflammation and viral replication at day 10 pi compared to WT BL/6 controls. (b) Representative histology sections of inflammation in WT and TRIF−/− hearts stained with H&E (top), magnification ×64. Data show the mean ± SEM of at least three separate experiments using 7 to 12 mice/group. **:  *P* < 0.01, ***:  *P* < 0.001.

Because TRIF participates in TLR3 and TLR4 signaling and TLR3 is known to protect against CVB3 myocarditis [14] while TLR4 increases disease [15], we were interested in determining whether TLR3- and TRIF-deficient mice developed similar disease. As expected, there was a significant increase in viral replication and acute myocarditis at day 10 pi in TLR3- or TRIF-deficient mice compared to WT controls (Figure 1), confirming previous reports [11, 14]. When we began these studies, only TLR3-deficient mice on a B6.129 background were available, and it was only very recently that Jackson Laboratories began to offer TLR3-deficient mice on a BL/6 background. Our preliminary studies with TLR3-deficient mice on a BL/6 background provide similar results as we found with TLR3-deficient B6.129 mice (data not shown), but we have not performed a complete analysis of BL/6 TLR3-deficient mice. Importantly, both BL/6 and B6.129 WT strains had a very similar level of inflammation and viral replication in the heart at day 10 pi (Figure 1). Based on these results, it appears that activation of TLR3 and TRIF inhibits CVB3 myocarditis in a similar manner.

### 3.2. Deficiency in TLR3 or TRIF Has a Different Effect on Survival

**Figure 2:**
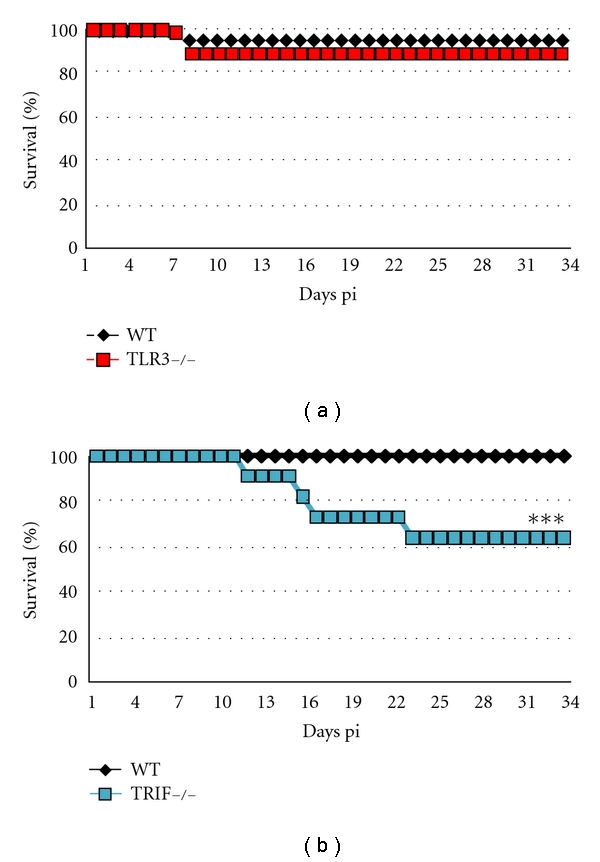
Survival differs between TLR3-deficient (TLR3−/−) and TRIF−/− mice with CVB3 myocarditis/DCM. (a) Survival of TLR3−/− mice compared to WT B6.129 controls (TLR3−/− versus WT, *n* = 77/group). (b) Survival of TRIF−/− mice compared to WT BL/6 controls (TRIF−/− versus WT, *n* = 45/group), ***:  *P* < 0.001.

Although the role of TLR3- or TRIF-deficiency appeared similar when examining the severity of viral replication and acute inflammation in the heart at day 10 pi (Figure 1), their effect on survival was distinctly different (Figure 2). Note that with this autoimmune CVB3 model of myocarditis nearly 100% of WT BL/6, B6.129, or BALB/c mice survive to day 35 pi [16, 17]. Most WT, and TLR3-deficient mice survived to day 35 pi (Figure 2). In contrast, 40% of TRIF-deficient mice died by day 25 pi (*P* < 0.001). These findings suggest that there are distinct differences in TLR3- versus TRIF-deficient mice that affect progression to chronic disease and HF.

### 3.3. TRIF-Deficient Mice Develop Significantly Worse Heart Function at Day 10 pi

**Figure 3:**
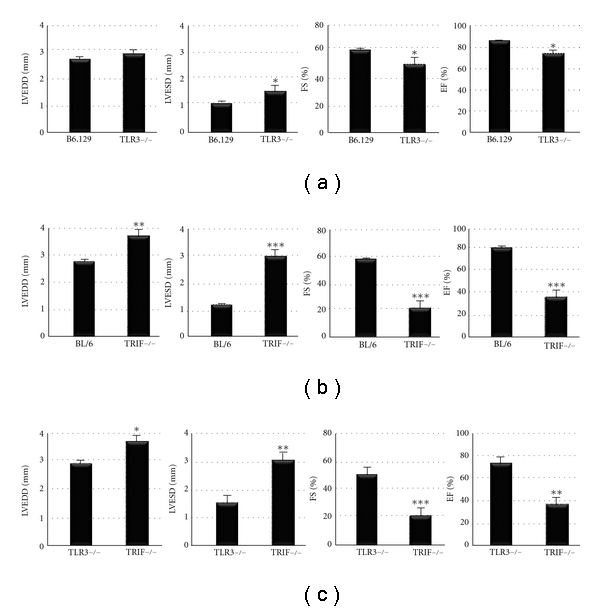
TRIF-deficient mice develop significantly worse heart function at day 10 pi. Echocardiography was used to assess heart function in (a) TLR3-deficient (TLR3−/−) versus WT B6.129 mice, (b) TRIF-deficient (TRIF−/−) versus WT BL/6 mice, or (c) TLR3- versus TRIF-deficient mice. LVEDD, left ventricular end diastolic dimension; LVESD, left ventricular end systolic dimension; FS, fractional shortening; EF, ejection fraction. Data show the mean ± SEM of at least three separate experiments using 7 to 12 mice/group. *:  *P* < 0.05; **:  *P* < 0.01; ***:  *P* < 0.001.

**Figure 4:**
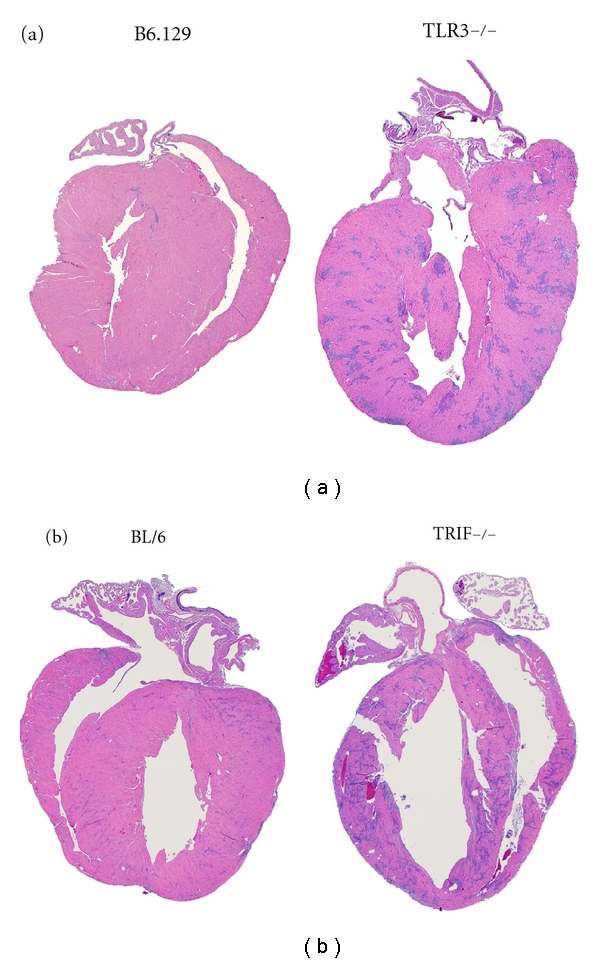
TRIF-deficient (TRIF−/−) mice develop DCM by day 10 pi, but TLR3−/− mice do not. Representative histology sections of (a) TLR3−/− compared to WT B6.129 controls at day 10 pi, magnification ×5. (b) Representative histology sections displaying dilation in TRIF−/− compared to WT BL/6 mice at day 10 pi, magnification ×5.

Next, we compared heart function in TLR3- versus TRIF-deficient mice during acute CVB3 myocarditis by echocardiography. We found that although TLR3- or TRIF-deficient mice developed significantly worse heart function compared to their WT controls (Figures 3(a) and 3(b), resp.), that TRIF-deficient mice were significantly worse compared to TLR3-deficient mice (Figure 3(c)). Importantly, there were no differences in heart function as assessed by echocardiography during acute CVB3 myocarditis between WT B6.129 or BL/6 strains (Figure 3). Echocardiography indicated that TRIF-deficient mice were already dilated by day 10 pi (see left ventricular end diastolic dimension/LVEDD) (Figure 3). Dilation in TRIF-deficient mice during acute CVB3 myocarditis was also observed by histology and is indicated by a thinning of the left ventricular wall in TRIF-deficient mice that was not observed in TLR3-deficient mice at day 10 pi (Figure 4). In susceptible mouse strains like BALB/c or A/J, dilation does not usually develop until day 35 pi, while B6.129 and BL/6 strains are resistant to the development of DCM in this model [16]. These findings indicate that TRIF is critically important in protecting against progression to DCM in resistant strains of mice like BL/6 and B6.129.

### 3.4. TLR3 versus TRIF Deficiency Affects Heart Function Differently during Acute and Chronic CVB3 Myocarditis

**Figure 5:**
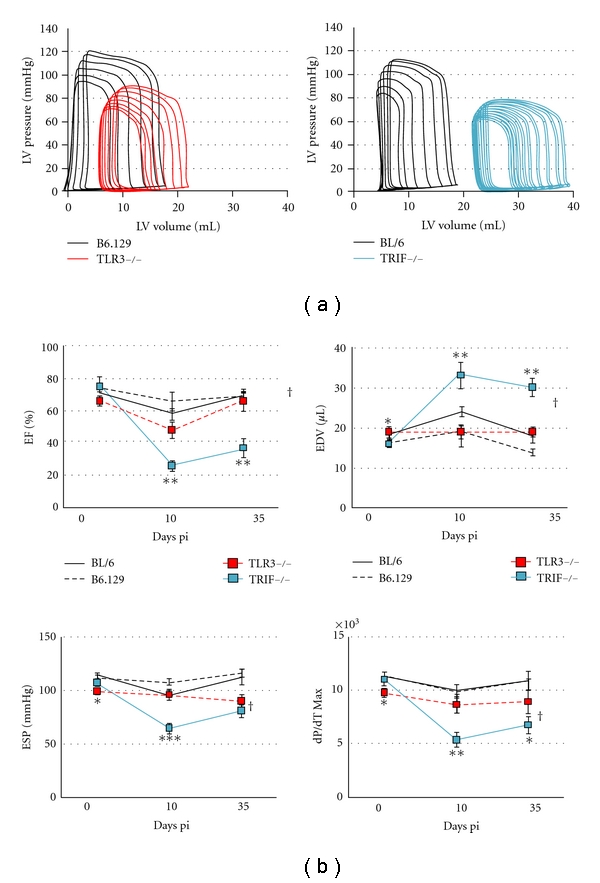
Cardiac function differs between TLR3 deficient (TLR3−/−) and TRIF−/− mice during acute and chronic CVB3 myocarditis. (a) Representative pressure-volume loops for TLR3−/− (left) and TRIF−/− (right) hearts at day 10 pi compared to their WT controls. (b) Comparison of pressure-volume relationships of TLR3−/−, TRIF−/− and WT mice at day 10 pi, *n* = 10 to 12 mice/group. Student's *t*-test compares TLR3−/− to TRIF−/− at day 0, 10 or 35 pi; *:  *P* < 0.05; **:  *P* < 0.01; ***:  *P* < 0.001. ANOVA compared TLR3−/− to TRIF−/− mice over time; ^†^, *P* < 0.05. EF: ejection fraction; dP/dT Max (mm Hg/s) measures the peak rate of pressure rise; ESP: end systolic pressure; EDV: end diastolic volume.

Further evidence that TLR3 and TRIF have separate roles in regulating the progression from myocarditis to DCM was obtained by comparing acute and chronic heart function using pressure-volume relationships in WT and knockout mice. Recall that B6.129 and BL/6 WT mice are resistant strains that do not develop the chronic phase of autoimmune CVB3 myocarditis and DCM [16]. We confirmed that these WT strains did not develop DCM in this study (Table 1 and Figure 5, see EDV day 35 pi versus day 0). No significant difference was observed in the severity of acute myocarditis between B6.129 and BL/6 WT mice at day 10 pi (Figure 1), or in their cardiac function prior to infection (day 0) or at day 10 and 35 pi for most parameters (Table 1 and Figure 5). Thus, cardiac function in WT B6.129 and BL/6 mice during acute and chronic CVB3 myocarditis is nearly identical (Table 1).

In contrast, a comparison of TLR3 to TRIF-deficient mice using pressure-volume relations demonstrated significantly diminished left ventricular (LV) function and increased dilation in TRIF-deficient mice at day 10 and 35 pi (Figure 5). End diastolic volume (EDV) is a measure of LV dilation and was significantly increased in TRIF-deficient mice by day 10 and 35 pi compared to TLR3-deficient mice, which were not dilated similar to WT B6.129 and BL/6 mice (Figure 5(b)). Thus, once TRIF-deficient mice became dilated at day 10 pi, they remained dilated to day 35 pi (Figure 5(b), see EDV). During acute myocarditis, ejection fraction (EF) was significantly lower in TRIF- than TLR3-deficient mice (26% ± 2.8 versus 48% ± 4.7, *P* < 0.05). An EF less than 40% indicates the risk for heart failure [22]. The peak rate of pressure rise (dP/dT Max) was significantly lower in TRIF-deficient mice compared to TLR3-deficient mice (5535 ± 688 versus 8803 ± 760 mmHg/s, *P* < 0.05). End systolic pressure (ESP) was significantly lower in TRIF- compared to TLR3-deficient mice during acute myocarditis (65 ± 4.5 versus 96 ± 4.7 mmHg, *P* < 0.05). Thus, many important functional parameters were significantly worse in TRIF- compared to TLR3-deficient mice by day 10 pi that persisted to day 35 pi, but these changes were not observed in WT mice (Figure 5 and Table 1). In fact, most functional parameters were not significantly different between BL/6 and B6.129 mice at baseline (day 0), day 10, or day 35 pi (Table 1). Thus, TRIF-deficient mice rapidly progressed to DCM and HF (e.g., low EF and reduced survival), while TLR3-deficient mice did not.

### 3.5. TLR3- and TRIF-Deficient Mice Differ in the Type of Th2 Response Induced during Acute Myocarditis

**Figure 6:**
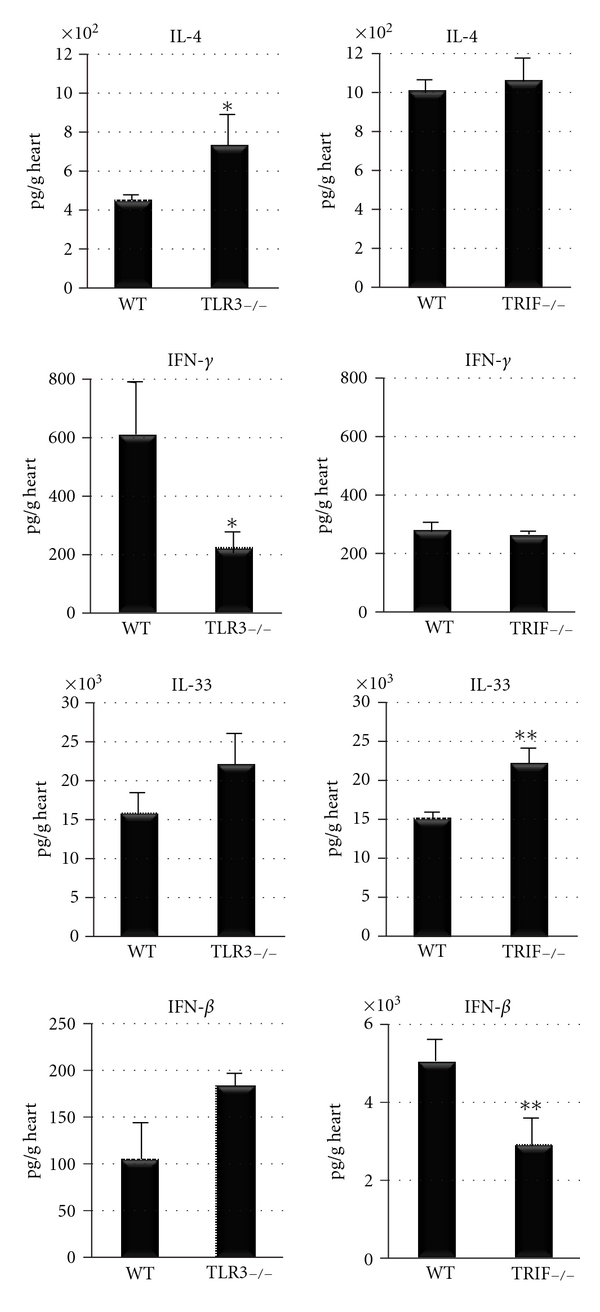
TLR3-deficient (TLR3−/−) mice have reduced IFN-*γ* and elevated IL-4 levels in the heart compared to WT B6.129 controls, while TRIF−/− mice have reduced IFN-*β* and increased IL-33 compared to WT BL/6 mice by ELISA. Similar results were obtained in at least three separate experiments and show the mean ± SEM of 7 to 12 mice/group. *:  *P* < 0.05; **:  *P* < 0.01.

Wild-type BL/6 and B6.129 mice characteristically produce Th1-type cytokines in response to infections or other stimuli. Because TLR3- and TRIF-deficient mice are known to have a defective Th1 response (i.e., reduced IFNs) following CVB3 infection [11, 14], we examined whether the Th1/Th2 balance had shifted in TLR3- and TRIF-deficient mice during acute myocarditis at day 10 pi by examining cytokine levels in the heart by ELISA. Previous reports had not described the type of Th2 response TLR3- or TRIF-deficient mice develop during CVB3 myocarditis [11, 14], and since susceptibility to chronic autoimmune myocarditis is dependent on a Th2-type immune response [16, 17], we were interested in determining whether a shift had occurred. We found that TLR3-deficient mice had significantly increased IL-4 (*P* = 0.03) and decreased IFN-*γ*  (*P* = 0.03) levels in the heart at day 10 pi (Figure 6), suggesting a shift to a Th2 response. In contrast, TRIF-deficient mice developed a different type of Th2 response characterized by significantly reduced IFN-*β* (a Th1 cytokine) (*P* = 0.009) and increased IL-33 (a Th2 cytokine) (*P* = 0.007) (Figure 6). To our knowledge, there are no reports describing that TLR3- or TRIF-deficient mice develop different types of Th2 responses.

Although the level of myocarditis, viral replication, and cardiac function can be compared between different experiments, a direct comparison of cytokines in WT mice should not be made because cytokine levels vary considerably between experiments (even in the same mouse strain) due to differences in processing the samples. So even though it appears as if BL/6 mice have higher levels of IL-4 and IFN-*β* during acute myocarditis (Figure 6, WT for TRIF−/−) compared to B6.129 mice (Figure 6, WT for TLR3−/−), this may only be an artifact of processing. We did not conduct an experiment to directly compare Th1 versus Th2 cytokine levels in WT BL/6 versus B6.129 mice. Thus, the only valid comparison for cytokine levels is the WT to the knockout. We consistently observed that TLR3-deficient mice developed significantly increased IL-4 and reduced IFN-*γ*, while TRIF-deficient mice developed significantly increased IL-33 and reduced IFN-*β*.

### 3.6. TLR3-Deficient Mice Have Increased Markers of Alternative Macrophage Activation during Acute Myocarditis

**Figure 7:**
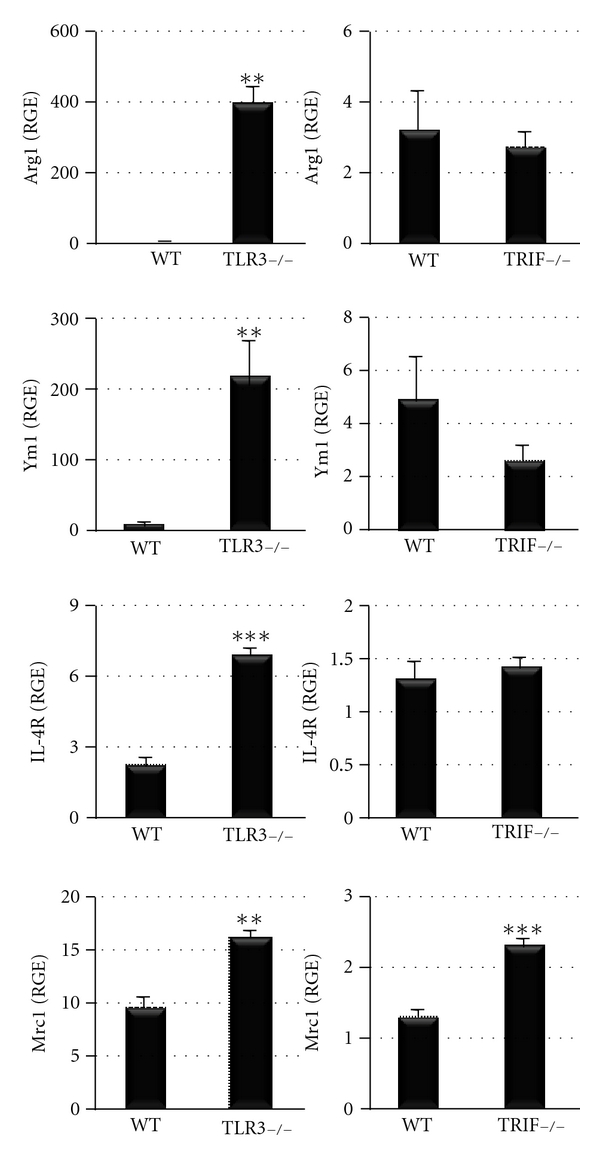
TLR3-deficient (TLR3−/−) mice have increased markers of IL-4-driven alternative activation compared to TRIF−/− mice. Markers of alternative activation included arginase-1 (Arg1), chitinase (Ym1), IL-4R, and macrophage mannose receptor (Mrc1) by qRT-PCR. Relative gene expression (RGE) was normalized to hypoxanthine phosphoribosyltransferase 1 (HPRT). Data show the mean ± SEM of 10 mice/group. **:  *P* < 0.01; ***:  *P* < 0.001.

To confirm that the increased IL-4 and decreased IFN-*γ* levels that we had observed in the heart of TLR3-deficient mice during acute myocarditis were due to a shift to a classic Th2 response, we examined markers of IL-4-driven alternative activation in the heart by qRT-PCR (Figure 7) [23, 24]. We found that arginase-1 (Arg-1), chitinase (Ym1), the IL-4 receptor (IL-4R), and the macrophage mannose receptor (Mrc1) were significantly increased in TLR3-deficient hearts compared to WT controls (Figure 7), indicating a switch to a Th2 response. Note the high-fold increase in Arg1 and Ym1 in TLR3-deficient hearts compared to WT controls or TRIF-deficient mice (Figure 7). In contrast, these classical markers of alternative activation were not increased in the heart of TRIF-deficient mice except for the macrophage mannose receptor (Figure 7). These data show that TLR3-deficient mice switch to a Th2 response during CVB3 myocarditis, while TRIF-deficient mice develop a distinctly different cytokine response involving the Th2-associated cytokine IL-33.

### 3.7. IL-33 Decreases Cardiac Function in WT BL/6 Mice

**Figure 8:**
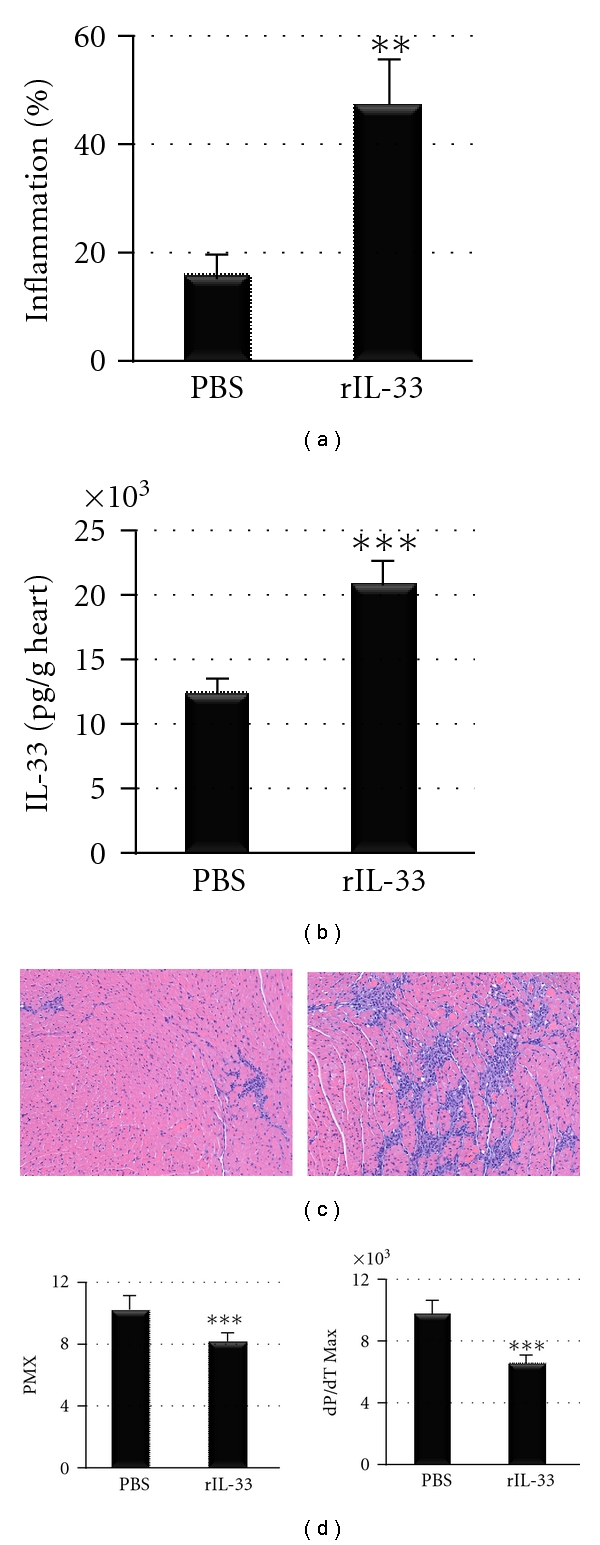
Recombinant IL-33 treatment increases myocarditis and cardiac dysfunction in BL/6 mice. Male BL/6 mice were treated with recombinant IL-33 (rIL-33) or PBS every other day from day 1 to 9 pi and (a,c) myocarditis, (b) cardiac IL-33 levels by ELISA and (d) heart function assessed using pressure-volume relationships at day 10 pi. (c) Representative histology sections of inflammation in PBS-treated (left) and rIL-33-treated (right) BL/6 mice stained with H&E, magnification ×64. Data show the mean ± SEM of 10 mice/group. **:  *P* < 0.01; ***:  *P* < 0.001.

Our data showed that although TRIF-deficient mice developed similar myocarditis compared to TLR3-deficient mice, they had significantly worse cardiac function. The elevation of IL-33 in TRIF-deficient hearts suggested that this cytokine could be responsible for increasing cardiac dysfunction in TRIF-deficient mice. We treated WT BL/6 mice with recombinant rIL-33 or PBS on day 1 through 9 pi following CVB3 infection on day 0 and examined heart function at day 10 pi. We found that rIL-33 treatment increased acute CVB3 myocarditis (Figures 8(a) and 8(c)), increased cardiac IL-33 levels (Figure 8(b)), and decreased cardiac function at day 10 pi (Figure 8(d)), indicating that elevated IL-33 in TRIF-deficient mice could lead to poor heart function in this strain.

### 3.8. IL-33 Improves Cardiac Dysfunction in TLR3-Deficient Mice during Acute Myocarditis

**Figure 9:**
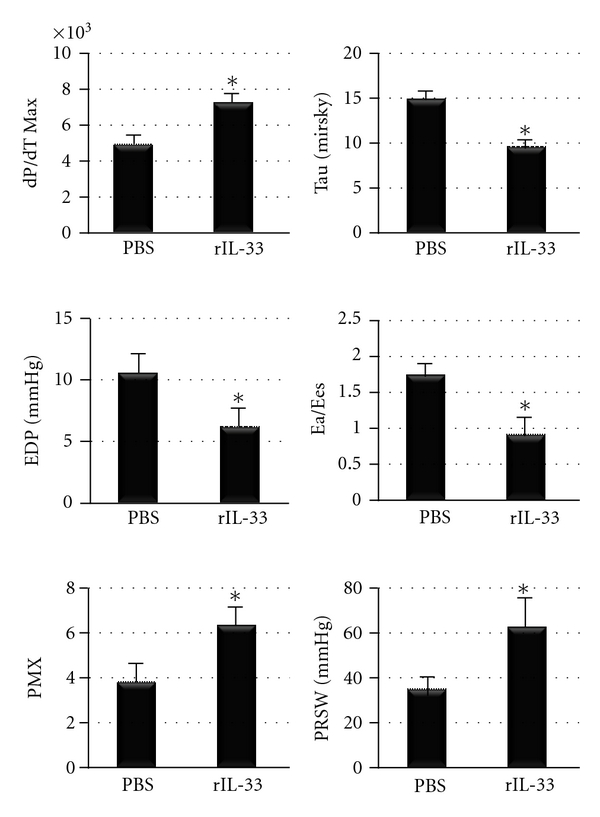
Summary of pressure-volume relationships at day 10 pi in TLR3−/− mice treated with recombinant IL-33 (rIL-33) or PBS every other day from day 1 to 9 pi. dP/dT Max measures the peak rate of pressure rise (mmHg/s); Tau, time constant of diastolic relaxation; EDP, end diastolic pressure; Ea/Ees, arterial elastance normalized to Ees which is left ventricular end systolic elastance (stiffness); PMX, maximum ventricular power; PRSW, preload recruitable stroke work. *n* = 10 to 12 mice/group. *:  *P* < 0.05.

Because IL-33-treatment was found to decrease cardiac function during acute CVB3 myocarditis in WT mice, we tested whether rIL-33 treatment of TLR3-deficient mice (that have a classic Th2 response) could worsen myocarditis. rIL-33 was administered ip to TLR3-deficient mice every other day from day 1 to 9 pi, as above, and heart function examined at day 10 pi. Surprisingly, pressure-volume analysis of heart function showed that rIL-33-treated TLR3-deficient mice had significantly improved heart function during acute CVB3 myocarditis (Figure 9, Table 2). Thus, in the context of a Th2-driven IL-4 response elevated IL-33 protects the heart from cardiac dysfunction during acute CVB3 myocarditis.

## 4. Discussion

Proinflammatory Th1 and Th17 immune responses are known to be important in the pathogenesis of experimental autoimmune myocarditis (EAM) and autoimmune CVB3 myocarditis mouse models [25–27]. Interestingly, Th2 responses have also been implicated in the pathogenesis of autoimmune myocarditis promoting DCM and HF [2, 17, 28, 29]. Only Th2-type responding susceptible mouse strains like BALB/c and A/J progress to chronic myocarditis and DCM in EAM and CVB3 myocarditis models [9, 16]. Additionally, IFN-*γ*-deficient mice, which have an elevated IL-4/Th2 response, develop severe DCM and HF following CVB3 myocarditis or EAM [17, 18]. Furthermore, IL-4 was found to increase EAM using rIL-4 treatment or anti-IL-4 antibodies [2, 28]. Evidence so far suggests that IL-4 increases autoimmune myocarditis by elevating autoantibody production and activating mast cells and alternatively activated macrophages that produce cytokines and enzymes needed for remodeling and fibrosis [2, 9, 17, 30–32].

In this study, TLR3-deficient mice had significantly increased markers of alternative activation and switched from IFN-*γ* to increased IL-4 production during acute CVB3 myocarditis, yet they did not develop severe DCM and HF. This suggests that an IL-4-driven Th2 response is not capable on its own of inducing HF, at least not in resistant strains of mice. Additionally, administration of rIL-33 to Th2-skewed TLR3-deficient mice reduced cardiac dysfunction during acute myocarditis. IL-33 is known to be able to induce Th2 responses on its own or with IL-4, but IL-33 is unique in that it can also increase proinflammatory Th1-type immune responses [33]. Our data show that if IL-33 is elevated when IL-4 is not also high, it has deleterious consequences on heart function. We are currently investigating the mechanism of action of IL-33, which is outside the scope of this paper. Although the mechanism has not yet been elucidated, the difference in cardiac function and survival/HF between TLR3- and TRIF-deficient mice suggests that TLR4-mediated TRIF may be responsible for reducing the negative cardiac effects of IL-33 in the heart during acute CVB3 myocarditis. We showed previously that more severe acute CVB3 myocarditis in male BALB/c mice is associated with elevated TLR4^+^  IL-1*β* ^+^ alternatively activated M2 macrophages in the heart [34]. Perhaps elevated IL-33 negatively impacts heart disease by increasing a proinflammatory M2 macrophage population in the heart leading to remodeling and HF. We are currently investigating this possibility.

Previous reports have found that increased myocarditis and HF in TLR3- or TRIF-deficient mice is due primarily to increased viral replication [11, 14]. However, this study suggests that high viral replication does not necessarily result in HF because TLR3-deficient mice had high levels of viral replication in the heart but were protected from severe chronic disease and HF. These findings suggest that additional factors beside direct viral damage are required for progression to DCM and HF such as elevated IL-33. IL-33 has been called an “alarmin” because it acts as a nuclear transcription factor until it is released from damaged cells when it acts as a cytokine via its receptor [35]. We show in this study that high viral replication does not itself account for increased IL-33 levels in the heart during CVB3 myocarditis because similar amounts of viral replication occurred in TLR3- and TRIF-deficient hearts, but IL-33 levels were only increased in TRIF-deficient mice. Because IL-33 is missing the signal peptide sequence required for secretion as a traditional cytokine [33], perhaps TRIF regulates the level of IL-33 within cells and then IL-33 is released by cellular damage due to viral replication thereby accounting for the differences in IL-33 levels between knockout strains.

The major limitation to this study is that TLR3- and TRIF-deficient mice are on a different background strain (TLR3−/− BL/6 mice were not available when we started these studies). Yet we provide evidence in this study that for all major parameters WT BL/6 and B6.129 strains responded in a similar manner during acute and chronic CVB3 myocarditis and are thus comparable backgrounds. This evidence includes (1) that both strains are “resistant” to the development of the chronic phase of CVB3 myocarditis and DCM (Figures 2 and 5, Table 1), (2) that both strains have almost identical heart function prior to infection (Table 1, day 0), (3) that both strains have nearly identical heart function as assessed by echocardiography and pressure-volume relationships during acute (day 10 pi) and chronic (day 35 pi) CVB3 myocarditis (Figures 3 and 5, Table 1), (4) that the severity and histologic appearance of acute myocarditis is the same for both strains (Figure 1), (5) that the level of viral replication in the heart during acute myocarditis is the same for both strains (Figure 1), and (6) that there is a similar level of M2 markers in the heart by RT-PCR for both WT strains (Figure 7, note difference in scale). However, it is possible that important differences exist between these backgrounds. We are currently examining this possibility by assessing TLR3-deficient mice on a BL/6 background.

In summary, we have shown in this study that elevated IL-4 or IL-4 plus IL-33 in the context of a Th2-driven response does not cause severe chronic disease resulting in HF. Yet outside of a classic IL-4-driven Th2-response, IL-33 is capable of causing cardiac dysfunction and HF as occurs in TRIF-deficient mice. Our findings suggest that the combination of high viral replication and elevated IL-33 levels in the heart is likely to be important in the progression from acute CVB3 myocarditis to severe DCM and HF. Our data also suggests that activation of TLR3 and TRIF in response to CVB3 infection protects Th1-responding mouse strains like BL/6 and B6.129 from progressing to chronic myocarditis, DCM, and HF. These findings provide insight into why most individuals that acquire CVB3 infection do not develop DCM and HF, and why polymorphisms in TLR3 signaling may predispose certain individuals to develop viral myocarditis, DCM, and HF.

## Figures and Tables

**Table 1:** Similarity in cardiac function of C57BL/6 versus B6.129 mice prior to infection (day 0) and during acute (day 10) or chronic (day 35) CVB3 myocarditis based on pressure-volume analysis.

	Day 0		Day 10 pi		Day 35 pi	
Parameter	BL/6	B6.129	BL/6	B6.129	BL/6	B6.129

HR	591 ± 3.9	596 ± 3.7	535 ± 6.0	567 ± 11.5*	569 ± 9.0	601 ± 5.7**
ESP	114 ± 2.1	111 ± 3.8	98 ± 2.1	108 ± 2.6**	109 ± 3.9	116 ± 3.4
EDP	6.3 ± 0.6	6.9 ± 2.5	4.1 ± 0.5	5.5 ± 0.5	4.8 ± 0.4	4.6 ± 0.7
dP/dT Max	11336 ± 463	10766 ± 250	10019 ± 538	10241 ± 395	11042 ± 852	10658 ± 555
dT/dT Min	−10628 ± 290	−10022 ± 309	−8484 ± 306	−9549 ± 454	−10017 ± 528	10852 ± 432
EF	71 ± 4.3	74 ± 2.2	58 ± 3.5	66 ± 5.7	69 ± 4.4	69 ± 2.4
ESV	5.7 ± 1.1	4.0 ± 0.4	10 ± 1.1	7.4 ± 1.8	6 ± 1.0	4 ± 0.2
EDV	18 ± 1.2	16 ± 1.0	24 ± 1.2	18 ± 2.5	18 ± 1.7	14 ± 0.7
CO	7499 ± 408	6940 ± 318	7276 ± 495	6083 ± 485	6885 ± 672	5960 ± 417
Ees	9.2 ± 0.8	8.3 ± 0.9	7.5 ± 0.5	8.4 ± 1.0	11.5 ± 1.2	15 ± 1.4
Ea/Ees	1.05 ± 0.1	1.3 ± 0.2	1.0 ± 0.0	1.1 ± 0.2	0.9 ± 0	0.8 ± 0.06
V_0_	−7.7 ± 1.3	−11.3 ± 1.6	−5.4 ± 1.2	−6.4 ± 2.1	−4.2 ± 1.9	−5.6 ± 1.0
Tau	5.2 ± 0.2	5.1 ± 0.3	5.6 ± 0.2	5.6 ± 0.2	5.6 ± 0.2	5.1 ± 0.2

CO (*μ*L/min), cardiac output; dP/dT Max, peak rate of pressure rise (mmHg/s); dP/dT Min, peak rate of pressure decline (mmHg/s); Ea/Ees, arterial elastance normalized to Ees; EDP (mmHg), end diastolic pressure; EDV (*μ*L), end diastolic volume; Ees (mmHg/*μ*L), LV end systolic elastance (stiffness); EF (%), ejection fraction; ESP (mmHg), end systolic pressure; ESV (*μ*L), end systolic volume; HR (bmp), heart rate; PFR/EDV (s^−1^), peak flow rate normalized to EDV; PMX/EDV^2^, maximum ventricular power normalized to EDV^2^ (mW/*μ*L^2^) × 100; PRSW (mmHg), preload recruitable stroke work; SV (*μ*L), stroke volume; SW, stroke work; Tau, Weiss (ms), time constant of diastolic relaxation; V_0_ (*μ*L), X-intercept of the ESP-volume relationship. *:*P* < 0.05, **:*P* < 0.01, and ***:*P* < 0.001 compare BL/6 to B6.129 by Student's *t*-test at each timepoint. Data shown as mean ± SEM for 10 mice/group per timepoint.

**Table 2:** *In vivo* hemodynamics of recombinant (r)IL-33-treated TLR3-deficient mice during acute CVB3 myocarditis (day 10 pi) based on pressure-volume analysis.

Parameter	PBS	rIL-33	*P* value
Heart rate	560 ± 12.0	555 ± 7.6	0.75
Developed pressure	66 ± 5.6	82 ± 3.9	0.04
EDP	11 ± 1.6	6 ± 0.8	0.04
dP/dT Max	4891 ± 537	7177 ± 523	0.01
dP/dT Min	−3597 ± 446	−6168 ± 850	0.02
EF	54 ± 4.0	60 ± 2.8	0.24
ESV	8 ± 1.6	5 ± 1.7	0.27
EDV	17 ± 1.9	14 ± 3.2	0.34
CO	5.0 ± 0.43	4.5 ± 0.96	0.58
PMX	4 ± 0.7	6 ± 0.7	0.03
PRSW	35 ± 6.0	62 ± 11.2	0.04
Ees	5 ± 0.4	14 ± 1.0	0.00001
E Max	13 ± 2.7	32 ± 3.9	0.002
Ea/Ees	1.7 ± 0.18	0.89 ± 0.19	0.01

CO, cardiac output (*μ*L/min); dP/dT max, peak rate of pressure rise (mmHg/s); dP/dT min, peak rate of pressure decline (mmHg/s); EDV, end diastolic volume (*μ*L); Ees, LV end systolic elastance; EF, ejection fraction (%); developed pressure, (ESP-EDP) (mmHg); ESV, end systolic volume (*μ*L); PRSW, preload recruitable stroke work; PMX, maximum ventricular power (mW); Ea/Ees, arterial elastance normalized to Ees; E Max, slope of line from end systole to end diastole. *P* values compare PBS-treated TLR3−/− to rIL-33-treated TLR3−/− by Student's *t*-test at day 10 pi. Data are shown as mean ± SEM for 11 to 12 mice/group. All mice were infected with CVB3 10 days prior to assessment.
